# General Notices

**Published:** 1893

**Authors:** 


### GENERAL NOTES.

### DOCTOR'S DON'T DISAGREE.

It may be recorded as an assured fact that the delegates to the Pan American Congress at Washington, who travel over the Chesapeake and Ohio Railway will with one accord praise its scenery and train service. There is nothing in the way of lovely mountain views and picturesque valleys to compare with the mountains and valleys of the Virginias through which the Chesapeake and Ohio Railway passes. There is nothing of historical interest in America as great as a trip through the Virginias, and there is no other railroad in America superior to the C. & O. in the smoothness and stability of its track and in the completeness and beauty of its trains, the F. F. V. Vestibule, Limited, being one of the famous trains of the world. The Chesapeake and Ohio Railway passes through Bull Run, Manassas, and other noted battlefields, and is in all respects the best route from the west, north-west and south-west to the national capital. For copy of "Virginia in Black and White," free, and for full information regarding rates and train service, address C. B. Ryan, Assistant General Passenger Agent, Cincinnati, Ohio.

Dr. E. J. Worst, of Ashland, Ohio, is credited with bringing out one of the greatest discoveries of the age, in his famous Australian Electro Pill remedy. It seems to act with magical effect upon the nervous system, similar to electricity, in which kidney, liver and stomach trouble and sympathetic diseases, yield immediately to its subtile treatment.

Dr. Worst agrees to mail each reader naming our paper, 7 days' treatment free. Address as above.

If you have gray hair and want to restore its natural color, Allen & Co., of Chicago, guarantee to do it for any of our readers. Also their remedy, Van's Mexican Hair Restorative will remove all dandruff, cures baldness, etc. Write them your wants.

Still, "Listerine" is heard from in all parts of the country. Dr. D. E. Ruff, of Junction City, Ore., writing regarding "Listerine," in treating cholera infantum, says: "I feel that my child's life was saved by the "Listerine," and since I first used it I have prescriced it in over one hundred cases and uniformly with success and happy results." Such testimonials speak for themselves.

Just at this season when rheumatic and nervous troubles come upon a person on account of the changeable weather, he wants to hie himself away to some retreat, and we cannot suggest any better place to go than Warsaw, N. Y., and enjoy for a season the treatment of the Warsaw Salt Baths and Sanitarium.

Do not be a sufferer from catarrh, when such a guarantee is presented as that of F. J. Cheney & Co., Toledo, Ohio. Their remedy, Hall's Catarrh Cure, is beyond a question, unequalled.

We want to call the attention, especially to the great Oriental Remedy, Herba Vita. This is the season of the year when you should prepare for the change of weather. This remedy is indispensable in the household. Many people would not be without it. Send for sample.

